# Overall Profile Measurements of Tiny Parts with Complicated Features with the Cradle-Type Five-Axis System

**DOI:** 10.3390/s21134609

**Published:** 2021-07-05

**Authors:** Lei Liu, Linlin Zhu, Li Miao, Chen Li, Changshuai Fang, Xiaodong Zhang

**Affiliations:** State Key Laboratory of Precision Measuring Technology & Instruments, Laboratory of Micro/Nano Manufacturing Technology, Tianjin University, Tianjin 300072, China; 3015202152@tju.edu.cn (L.L.); l_linzhu@tju.edu.cn (L.Z.); miaoliii@tju.edu.cn (L.M.); 2019202247@tju.edu.cn (C.L.); cshfang@tju.edu.cn (C.F.)

**Keywords:** five-axis, cradle type, point-scanning, error modeling

## Abstract

There are generally complex features with large curvature or narrow space on surfaces of complicated tiny parts, which makes high-precision measurements of their three-dimensional (3D) overall profiles a long-lasting industrial problem. This paper proposes a feasible measurement solution to this problem, by designing a cradle-type point-scanning five-axis measurement system. All the key technology of this system is also studied from the system construction to the actual measurement process, and the measurement accuracy is improved through error calibration and compensation. Finally, the feasibility is proved by engineering realization. The measurement capability of the system is verified by measuring workpieces such as cross cylinders and microtriangular pyramids.

## 1. Introduction

The entire sizes of tiny parts with complicated features are in the millimeter or centimeter level, and they generally have typical structural features such as large curvature surfaces, narrow areas, complex structures, sharp edges, etc. [1]. Tiny parts such as crossed cylinders, diamond cutters, microtriangular pyramids, etc., have been widely used in many fields such as aerospace, biomedicine, telecommunication, intelligent manufacturing, and optical communication [2,3]. The application performance of tiny parts is affected by the manufacture quality of their surface profiles, so it is of great significance to focus on their surface profile measurements [4,5]. One of the research hotspots in the measurement field of tiny parts is to measure their typical structures. The overall profile measurement is to measure the complete surface profiles of workpieces without blind spots and obtain 3D profile information. To realize these measurements, measurement systems with multiple motion axes are required, which are also called MDOF (multi-degree-of-freedom) measurement systems [6]. MDOF systems can flexibly adjust the relative postures and positions between probes and workpieces through multiaxis linkage motions to achieve scanning measurements on whole surfaces [7]. However, the measurement accuracy of systems is a composite indicator of the accuracy of sensors and multiaxis linkage mechanisms [8,9]. While MDOF measurement systems increase the flexibility of motions, they also introduce more errors and error coupling relationships [10,11]. These errors make high-precision measurements with MDOF measurement systems become a long-lasting problem in industry.

Currently, two scanning motion forms, namely, the rotary motions of probes or workpieces are mainly used for profile measurements from multiple relative positions. As the source of surface data of measured workpieces, different probes cover a variety of different measurement principles and forms. Generally, Line-scanning and local-surface-scanning measurement forms are limited in dynamic range among existing measurement methods, and need point cloud registration to achieve large-scale surface reconstruction. In contrast, measurement forms based on single-point probes have the largest dynamic range and highest flexibility, and can achieve 3D measurements of any surfaces with complex features through point scanning [12]. Among them, non-contact point-scanning measurement principles are more efficient than traditional contact CMMs or similar instruments and will not scratch measured surfaces [13]. Firstly, as for MDOF measurement systems with rotary point-scanning probes, there have been several commercial instruments, among which Nanomefos by Dutch Optics Centre and Demcon [14] and Luphoscan by Taylor Hobson [15] are the most typical. However, the measurement accuracy of these instruments depends on high-precision feedback mechanisms such as laser interferometers [16], so these systems are relatively complex and expensive, and cannot be widely used in industrial applications. Besides, Luphoscan cannot measure most non-rotational asymmetric optical free-form components due to its structure. To solve this problem, our research group have designed and built a five-axis point-scanning measurement system, which controls the probe to rotate in two dimensions so that the probe can maintain postures along normal vectors of measured surfaces [17]. After studying the technology of system error modeling and compensation, high-precision measurements of large-curvature optical free-form surfaces are realized. However, the rotary radii of probes are relatively large in MDOF measurement systems, which will result in the waste of space and travels of motion mechanisms. The systems mentioned above can measure part of the area of whole surfaces on optical components within a certain angle range, but still cannot realize the entire surface measurements, let alone the entire surfaces of complicated tiny parts. Secondly, as for rotation scanning by workpieces, most measurement systems only use the single rotary stage to adjust workpieces, and few use the form of dual-axis rotation, such as S Neox 3D optical profiler by Sensofar [18] and StentCheck 3D CMM by Werth [19]. The S Neox 3D optical profiler uses an AC dual-axis rotary stage (cradle-type structure [20,21]) similar with some five-axis machining centers to adjust the posture of workpieces. This instrument has the capability to measure entire surfaces of milling cutters. Since its probes are based on the local-surface-scanning measurement principle, point cloud registration is needed, which destroys the continuity of measurements and reduces the measurement accuracy. The StentCheck 3D CMM uses a tilting table and rotation stage to rotate measured workpieces in two dimensions, and its efficiency is much higher than that of the S Neox 3D optical profiler. However, the measurement range of this system is limited by the angle range of the tilting table, and it cannot realize the whole surface scanning of tiny parts with more complicated structures. However, the cradle-type structure for workpiece rotation has certain advantages. Compared with rotating probes, only rotating measured tiny parts can achieve a wide range of angular movements in space, and the radii of rotations are relatively small. The cradle-type structure makes the posture adjustment more accurate and efficient, and saves space and the movement of motion mechanisms [22]. It can be seen that the method of rotating workpieces on a cradle-type five-axis system is a relatively promising measurement solution, which is worthy of further study.

The core problem that restricts the development of high-precision point-scanning measurement systems for a long time is the measurement accuracy, which highly depends on the accuracy of system hardware. The introduction of multiaxis motion mechanisms has increased the scanning motion errors of MDOF measurement systems [23]. Therefore, based on certain hardware conditions, there is an urgent need for research on high-precision system error calibration and compensation to improve the measurement accuracy of these systems. Most research on the error modeling and compensation of MDOF systems focuses on CNC machine tools currently [24]. Based on the multibody kinematics theory, Zhang Y. et al. deduced a set of transformation formulas for cradle-type five-axis CNC machine tools, but their study only covers theoretical models without actual experiments or error compensation research [25]. Schwenke H. et al. used a laser interferometer for the error calibration of CNC machine tools and analyzed each rotary axis in 6 degrees of freedom [26]. Chen J et al. put forward a method to calibrate errors of rotary stages on CNC machine tools by using a double ball bar system as the calibration part [27]. In summary, on the one hand, most methods for the error modeling of MDOF systems need high-precision instruments for calibration, which limits the flexibility and practicality of methods [28]; on the other hand, these methods are mostly aimed at CNC machine tools rather than measurement systems [29,30]. Therefore, to solve the common problem of large measurement errors faced by MDOF measurement systems, suitable mathematical analysis, error modeling, error calibration, and compensation methods are required.

In this paper, focusing on the problem of the overall profile measurements of tiny parts with complicated features, we propose a feasible solution with the cradle-style five-axis point-scanning measurement system, and achieve the engineering realization. The key technology from system construction, path planning, system error modeling, and compensation to actual measurement process was studied. The five-axis cradle-type system built in this paper controls the probe to rotate in space to realize scanning measurements on whole surfaces of measured workpieces without blind spots. This system realizes the real-time tracking of measured points through the coordinate recursive algorithm, so point cloud registration is not needed, which ensures the continuity of scanning measurement and improves measurement efficiency. Focusing on the improvement of the accuracy of this point-scanning measurement system, procedures, and related algorithms of system error identification, calibration, and compensation are proposed. The accuracy is ensured by error correction from the source, so it does not rely too much on high-precision feedback mechanisms. The advantage of error correction method we proposed is that it improves the accuracy of the measurement system conveniently and effectively without the need for additional high-precision instruments. Finally, the measurement capability and accuracy of our system are verified by measuring cross cylinders, standard spheres, and other workpieces.

## 2. Measurement Scheme

**Figure 1:**
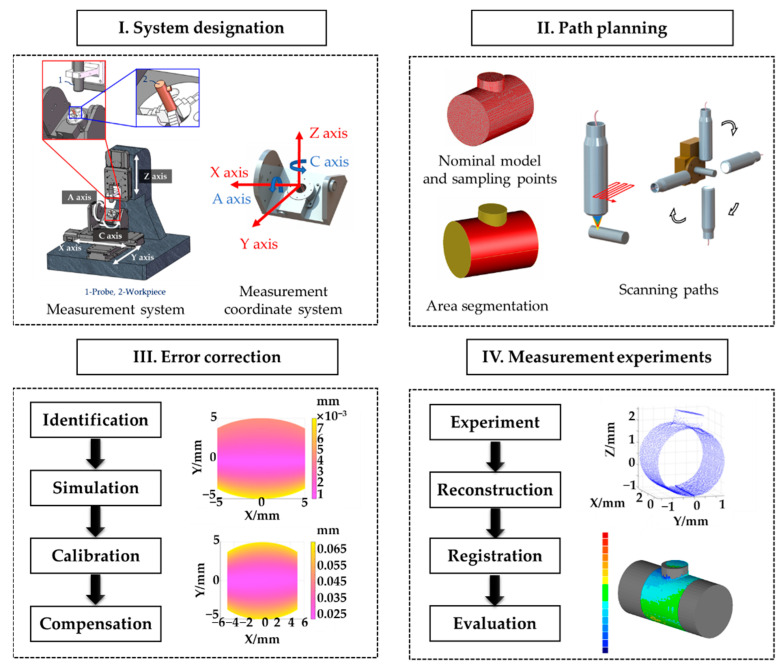
A schematic diagram for overall 3D profile measurements of tiny parts.

The general idea of this paper is shown in Figure 1. A cradle-type five-axis point-scanning measurement system structure was designed to measure the overall 3D profiles of tiny parts. By analyzing the relationship between axes in this system, a model of measurement coordinate system was constructed based on the kinematic theory, meanwhile coordinates of real-time measuring points were calculated. For specific measured workpieces, it is necessary to design measurement paths based on their surfaces. Nominal models of measured workpieces were analyzed, and their overall surfaces were segmented into several areas. Scanning paths were designed according to specific features. Compared with traditional three-axis or four-axis measurement systems, complicated correlation among axes in five-axis systems introduced more error terms. Error terms of this five-axis system were identified, classified according to their influence mechanisms, and their impacts on measurement results were simulated. To improve efficiency, only major error terms (which account for most of the proportion to measurement accuracy) were concerned. A calibration and compensation process for major error terms was proposed and verified via simulation to test theory feasibility. An experiment setup was built and calibrated based on theorical research. Four kinds of tiny parts were measured and reconstructed with point clouds. The registration between point clouds and nominal models was applied to evaluate measurement accuracy.

## 3. Coordinate System Construction and Path Planning

The cradle-type five-axis measurement system mainly consists of electric motion stages and a probe. This system has three linear axes of X, Y, and Z, two rotary axes of A and C. To reduce positioning errors caused by the movement of neighboring axes, this self-built system separates the Z axis from the X axis and Y axis. The cradle-type due-axis setup consists of A-axis and C-axis rotary stages, which are orthogonal. Measured workpieces are installed on the C-axis rotary stage by a three-jaw chuck. The spatial position and orientation of workpieces are adjusted by four motion axes, while the position of the confocal probe is only adjusted by the Z-axis linear stage. Actually, the basic idea of this paper can be straightforwardly extended to any configurations of five-axis system. The point-scanning probe used is a chromatic confocal probe, which has significant advantages on strong anti-interference and can realize high-precision measurement of workpieces.

**Figure 2:**
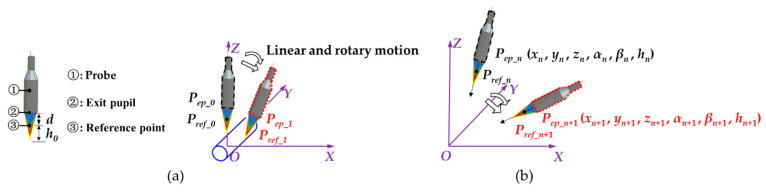
Schematic diagrams for coordinates recursion: (**a**) calculate the first point coordinates; (**b**) calculate the *n* + 1th point coordinates.

Figure 2 shows the process of coordinates recursion. The coordinate system, following the measurement system model, is a right-hand coordinate system. The directions of axes are the same as the nominal directions of linear stages or rotary stages. When analyzing the motion trajectory of coordinate points with the idea of relative motion, the measured workpieces can be considered as fixed, while the probe performs all the motions. Thus, the situation that workpieces rotate around the A axis and C axis is regarded as the probe rotating around the A axis and C axis in opposite directions. The spatial coordinates of point cloud were calculated with measurement data of different measurement areas and paths, and they were all summarized in this measurement coordinate system to restore the overall 3D profile.

A standard cylinder of a known diameter was used to show the principle of the coordinate calculation. Since chromatic confocal probes can only measure relative distances, that means, measurement data is the distance along the optical axis between the measuring point and a reference plane. The reference point is the intersection point between the optical axis and the reference plane, and the working distance is the distance between the exit pupil of the probe and its reference point. Suppose the working distance of the probe is *d*, the radius of the standard cylinder is *r*, and the initial measurement result is *h*_0_.

The process of calculating coordinates of the first measuring point in the measurement coordinate system is shown in Figure 2a. The initial coordinates ***P_ep__*_0_** of the exit pupil are (0, 0, *d* + *r* + *h*_0_), and the initial coordinates ***P_ref__*_0_** of the reference point are (0, 0, *r* + *h*_0_). The measurement data transmitted from the measurement system to the computer at a time includes six terms (*x_i_*, *y_i_*, *z_i_*, *α*, *β*, and *h_i_*), which represent the real-time position information of the X, Y, Z, A, and C axes, and the measurement data of the chromatic confocal probe.

Due to the motions of three linear stages, the position of the exit pupil of the probe will change from (*0*, *0*, *d + r+ h*_0_) to (*x*_1_, *y*_1_, *z*_1_ *+ d + r + h*_0_), and the reference point will move to (*x*_1_, *y*_1_, *z*_1_ *+ r + h*_0_). In three-dimensional space, each rotary axis can be positioned by two points on it, which is named *c*_1_ (*a*, *b*, *c*) and *c*_2_. According to the rotation transformation principle of rigid body in three dimensions, the vector of the axis ***p*** and rotary matrix ***R*** can be written as follows:

(1) $$p=\left[\begin{array}{c} u\\ v\\ w \end{array}\right]=\frac{c_{2}-c_{1}}{norm(c_{2}-c_{1})}$$

(2) $$R=\left[\begin{array}{cccc} u^{2}+(v^{2}+w^{2})\cos(\gamma ) & uv(1-\cos(\gamma ))-w\sin(\gamma ) & uw(1-\cos(\gamma ))+v\sin(\gamma ) & \left(a(v^{2}+w^{2})-u(bv+cw))(1-\cos(\gamma ))+(bw-cv)\sin(\gamma \right)\\ uv(1-\cos(\gamma ))+w\sin(\gamma ) & v^{2}+(u^{2}+w^{2})\cos(\gamma ) & vw(1-\cos(\gamma ))-u\sin(\gamma ) & \left(b(u^{2}+w^{2})-v(au+cw))(1-\cos(\gamma ))+(cu-aw)\sin(\gamma \right)\\ uw(1-\cos(\gamma ))-v\sin(\gamma ) & vw(1-\cos(\gamma ))+u\sin(\gamma ) & w^{2}+(u^{2}+v^{2})\cos(\gamma ) & \left(c(u^{2}+v^{2})-w(au+bv))(1-\cos(\gamma ))+(av-bu)\sin(\gamma \right)\\ 0 & 0 & 0 & 1 \end{array}\right]$$

After rotations, the coordinates of the exit pupil of the probe ***P_ep__*****_1_** and reference point ***P_ref__*_1_** are expressed as Equations (3) and (4).

(3) $$P_{ep\_1}=R_{A\_1}\times R_{C\_1}\times \left[\begin{array}{c} x_{1}\\ y_{1}\\ z_{1}+d+r+h_{0}\\ 1 \end{array}\right]$$

(4) $$P_{ref\_1}=R_{A\_1}\times R_{C\_1}\times \left[\begin{array}{c} x_{1}\\ y_{1}\\ z_{1}+r+h_{0}\\ 1 \end{array}\right]$$

The optical axis vector ***m*** can indicate the spatial orientation of the probe, and can be calculated according to the current positions of the exit pupil of the probe and reference point:

(5) $$m=(P_{ep\_1}-P_{ref\_1})/norm(P_{ep\_1}-P_{ref\_1})$$

Combined with the measurement result *h*_1_, the spatial coordinates of the first measuring point in the global measurement coordinate system ***P_point__*_1_** can be calculated as Equation (6).

(6) $$P_{point\_1}=P_{ref\_1}+h_{1}\times m$$

As shown in Figure 2b, every time when measuring the next point, the data of five motion axes recorded at the latest point were used as a reference to calculate the increment motions of each axis. Based on the data (*x_n_*, *y_n_*, *z_n_*, *α_n_*, and *β_n_*) at the *n*th measuring point and the data (*x_n+_*_1_, *y_n+_*_1_, *z_n+_*_1_, *α_n+_*_1_, *β_n+_*_1_, and *h_n+_*_1_) obtained at the *n +* 1th measuring point, the process of calculating the coordinates *P_point_n_*_+1_ of the *n* + 1th point was as follows:

Calculate the increment motions *x_add_*, *y_add_*, *z_add_*, *α_add_*, and *β_add_* of the X, Y, Z, A, and C axes, respectively. Due to the cradle-type structure, rotations around the C axis change the directions of linear motions by X, Y, and Z stages, while rotations around the A axis change the directions of X, Y, and Z stages and the vector of the C axis. Each motion is recorded, and these vectors were calculated. The translation matrix ***T****_i_* is expressed as Equation (7), where ***axis****_x_i_*, ***axis****_y_i_*, and ***axis****_z_i_* are the vectors of X, Y, and Z stages, and ***O*** is a 3 × 3 matrix of zeros.

(7) $$T_{i}=\left[\begin{array}{cccc} axis_{x\_i} & axis_{y\_i} & axis_{z\_i} & O\\ 0 & 0 & 0 & 1 \end{array}\right]$$

Equations (8) and (9) were used to express the coordinates of the exit pupil ***P_ep_****_**_n+_*****_1_** and reference point ***P_ref_****_**_n+_*****_1_** during the *n* + 1th measurement.

(8) $$P_{ep\_n+1}=R_{A\_n+1}\times R_{C\_n+1}\times (P_{ep\_n}+T_{i}\times \left[\begin{array}{c} x_{add}\\ y_{add}\\ z_{add}\\ 1 \end{array}\right])$$

(9) $$P_{ref\_n+1}=R_{A\_n+1}\times R_{C\_n+1}\times (P_{ref\_n}+T_{i}\times \left[\begin{array}{c} x_{add}\\ y_{add}\\ z_{add}\\ 1 \end{array}\right])$$

where, ***P_ep___n_*** and ***P_ref___n_*** represent the coordinates of the exit pupil and reference point during the *n*th measurement.

Similarly, the optical axis vector was calculated according to the coordinates of the pupil and reference point, and the coordinates of the *n* + 1th measuring point were calculated in combination with the measurement result *h_n+_*_1_. In summary, based on the iterative theory, the spatial coordinates of all measured points can be derived. All points were on the scanning paths and coordinates were calculated in the same measurement coordinate system recursively. Point cloud registration was not needed because the relative position of these points was consistent with the actual situation.

Since the chromatic confocal probe is a point measurement probe, it is necessary to design scanning paths according to certain measured surfaces. Limited by the angular characteristics and range of the probe, the contour lines with a larger curvature are usually regarded as boundaries of different measurement areas, and scanning paths are designed based on them. The surface near contour lines is scanned by special paths according to its normal vectors. After area-by-area measurements, the coordinates of the point cloud can be summarized.

**Figure 3:**
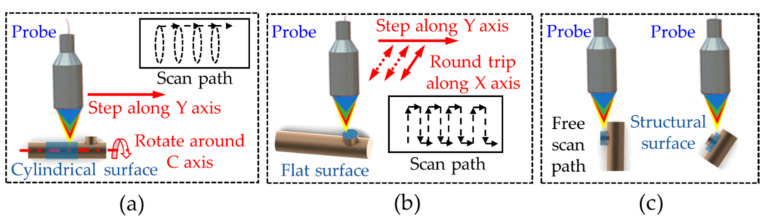
Schematic diagrams for different measurement paths: (**a**) rotary scanning paths; (**b**) raster paths; (**c**) free scanning paths.

There are usually three common types of profile features of tiny parts: cylindrical surface, flat surface, and complicated structure surface. As shown in Figure 3, three measurement paths were proposed for these three types of surfaces. Rotary scanning paths are suitable for measuring the surfaces whose overall contours are in the form of rotation. The Y-axis motions are periodic steps, and the C-axis rotary stage controls workpieces to rotate 360° to achieve a fixed interval point measurement on every circular path. Raster paths are suitable for relatively flat surfaces, whose height change in a small range. Raster paths are conventional for numerical-control machine applications. Free scanning paths are suitable for measuring more complicated surfaces, such as area near contour lines, and depending on the normal vectors of specific measured features, the relative spatial position of the probe and workpieces need to be adjusted appropriately to meet the angle characteristics of the probe. When measuring each workpiece, the paths of different areas are generated separately. However, the measurement path is continuous, so the measurement is also a continuous process.

## 4. Error Correction Theory of the Cradle-Type Measurement System

### 4.1. Error Identification

Compared with traditional three-axis or four-axis systems, with the increased number of synchronous motion axes, there are more error terms and error coupling relations in five-axis systems. These error terms cause great harm to systems’ accuracy, especially for point-scanning systems and CNC machine tools [31]. Actually, the most critical problem that point-scanning measurement systems need to solve is the calibration and error compensation of motion mechanisms.

**Figure 4:**
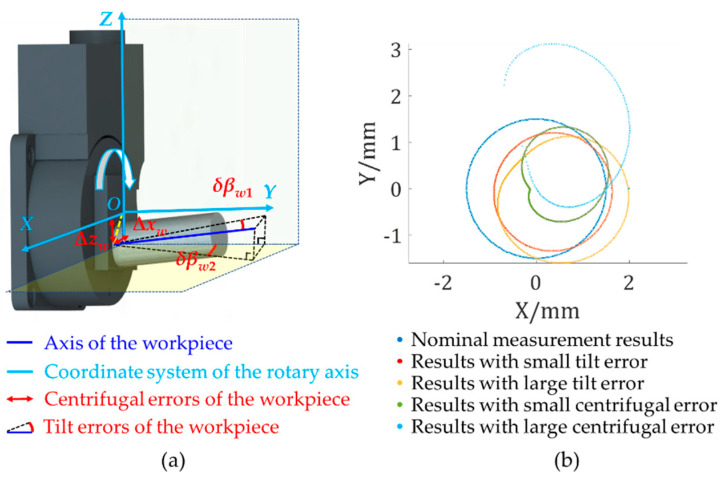
Error identification and simulation: (**a**) a schematic diagram for clamping errors of workpieces; (**b**) simulation results on clamping errors.

As shown in Table 1, to facilitate error calibration and compensation, the error terms in five-axis systems can be divided into two types: system errors and clamping errors of workpieces. System errors can be divided into four types, namely the static errors of the linear stage (*δx*, *δy*, and *δz*), static errors of the rotary stage (*δθ*_1_, *δθ*_2_, Δ*x*, Δ*y*, and Δ*z*), dynamic errors of the linear stage (Δ*d*), and dynamic errors of the rotary stage (*δβ*). The first two belong to static system errors, which stem from the inaccurate clamping and is fixed when the system is built, and the latter two belong to dynamic system errors, which stem from motions of the linear or rotary axes and are random. The clamping errors of measured workpieces are divided into tilt errors (*δβ_w_*_1_ and *δβ_w_*_2_) and centrifugal errors (Δ*x_w_* and Δ*z_w_*), and both are static errors. The schematic diagram of clamping errors is shown in Figure 4a, with a cylinder as the workpiece. Cylinders have central axes, which can indicate the degrees in which workpieces are usually tilted. The axis of the workpiece may not be perpendicular to the rotary stage surface, so there is a certain angle between this axis and the axis of the stage. Tilt errors are the angles between the projection of the axis of the measured workpiece and coordinate axes in the plane, which is perpendicular to the C axis. Although the axis of the workpiece is parallel to the C axis, they are not coincident. The distance between two parallel axes represents the degree of deviation of two axes, and the two orthogonal components of this distance in the plane perpendicular to the C axis are called centrifugal errors.

Based on the kinematic theory [32], the measurement system can be divided into the workpiece chain and probe chain. The proportion of influence on results from different error terms was calculated as follows: The measurement results can be written as a multivariate function formula with all error terms. All values of error terms were set based on the real situation, that is, angular error values of rotary stages were in the range of 0.01–0.02°, and linear error values of linear stages were in the range of 0–1 μm. The default tilt error values of workpieces were 0–5°, and the centrifugal error values were 0–0.5 mm. Ratios are shown in Table 1. Among all errors, static errors of rotary stages and clamping errors had a larger proportion than others. In a cradle-type five-axis system, the static errors of rotary stages and clamping errors accounted for about 97.12% of the influence on the results, so they were defined as the major error terms in this paper. Besides, the simulation results of measuring a cross-sectional profile of a standard cylinder with clamping errors are shown in Figure 4b, and even small clamping errors will cause a large measurement deviation.

### 4.2. Error Calibration and Compensation

In Section 4.1, major error terms were identified. To improve calibration efficiency and avoid more errors caused by the calibration process when calibrating multiple error terms, only major error terms (the static errors of rotary stages and clamping errors of workpieces) were selected for calibration and compensation. A standard cylinder was used as the calibration part in calibration. The standard cylinder was rotationally symmetric. Compared with the standard sphere, it can provide a certain rotary axis as a reference, so it is more suitable for determining the direction vector in space and the rotary axis of a rotary stage. The process of correcting the major error terms is as follows: (a) calibrate and compensate the static errors of the A-axis rotary stage first; (b) calibrate and compensate the static errors of the C-axis rotary stage; (c) calibrate and compensate the clamping errors of the measured workpiece.

#### 4.2.1. Calibration and Compensation of Static Errors of Rotary Stages

**Figure 5:**
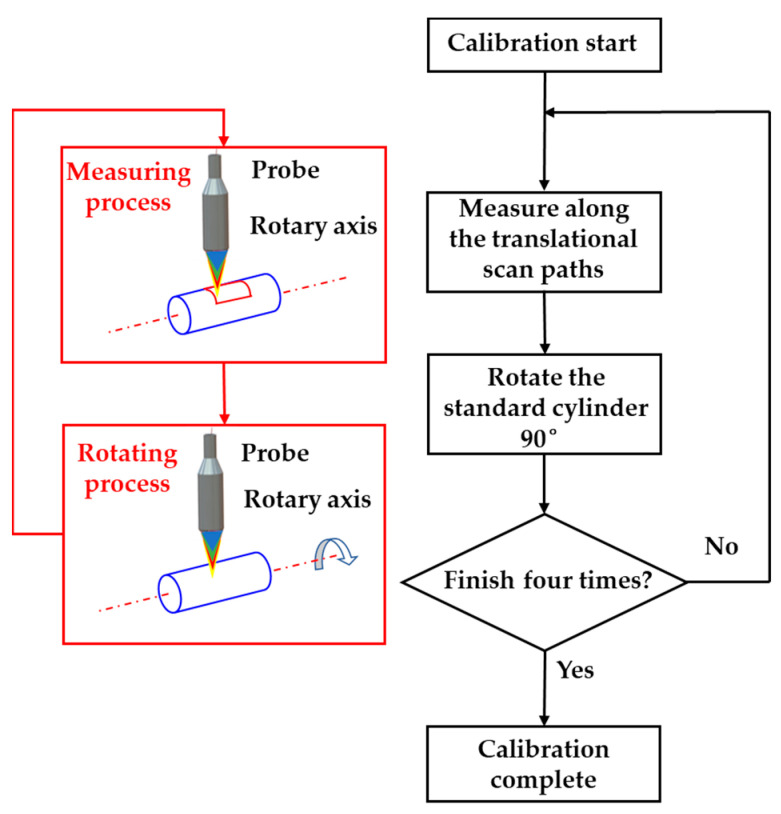
The calibration process of the static errors of a rotary stage.

The static errors of two rotary stages were calibrated and compensated at first, and the method was the same. As shown in Figure 5, when calibrating the static errors of the rotary stage, a standard cylinder was clamped on it and rotated 0°, 90°, 180°, and 270° respectively, and a piece of area was scanned at these four angles. Since four point clouds were the minimum amount to form symmetry in two directions, the stable angle between two point clouds was 90°.

The rotary axes of the cylinder at these four positions could be calculated by cylindrical fitting with the measurement results, named ***L***_1_, ***L***_2_, ***L***_3_, and ***L***_4_, and the other three axes were generated by the rotation of the first axis ***L***_1_. Four axes were symmetrically distributed around the rotary axis of the rotary stage, so that the real vector of it could be calculated by optimization. The coordinates of the two points on the rotary axis were the optimization objects. The first axis ***L***_1_ will be rotated 90°, 180°, and 270° around the optimized rotary axis and the form ***L***_2′_, ***L***_3′_, ***L***_4′_, ***L***_2′_, ***L***_3′_, and ***L***_4′_ did not coincide with ***L***_2_, ***L***_3_, and ***L***_4_, and the optimization was finished when the total distance between them reached the minimum.

**Figure 6:**
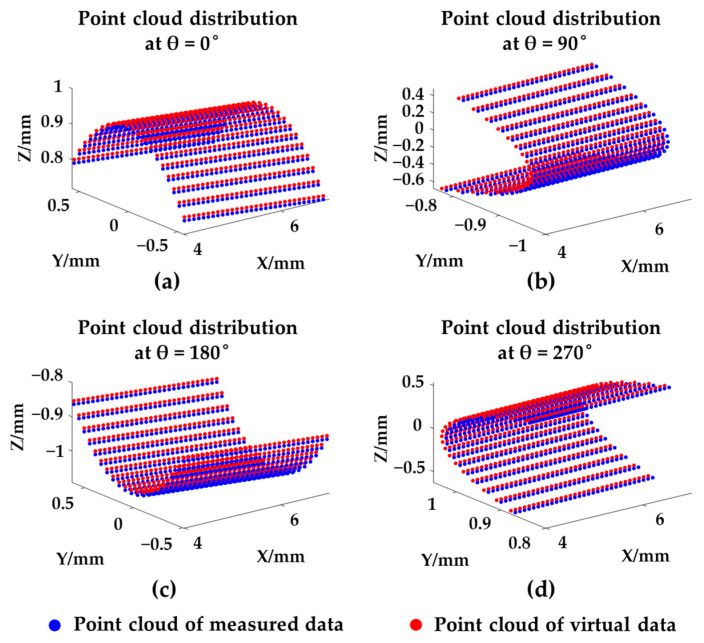
The point cloud distribution of the rotary axis calibration in simulation: (**a**) θ = 0°; (**b**) θ = 90°; (**c**) θ = 180°; (**d**) θ = 270°.

By taking the optimized vector as the rotary axis, the first point cloud at 0° was rotated 0°, 90°, 180°, and 270° around it and four virtual point clouds were generated. As shown in Figure 6, both the measured and virtual point clouds were compared in the same coordinate system, which were drawn in blue and red, respectively.

The coordinate system O***^’^***-X***^’^***Y***^’^***Z***^’^*** of the rotary stage did not coincide with the measurement coordinate system O-XYZ. Coordinates of the direction vectors ***O^’^X^’^***, ***O^’^Y^’^***, ***O^’^Z^’^,*** and the origin ***O^’^*** in O-XYZ can be expressed according to the static errors of the rotary stage. By using the coordinate space transformation matrices, the coordinates of the measured point ***P^’^*** can be converted to the coordinates ***P*** in the global measurement coordinate system:

(10) $$P=\left[\begin{array}{c} \begin{array}{cccc} P_{x} & P_{y} & P_{z} & P_{o} \end{array}\\ \begin{array}{cccc} 0 & 0 & 0 & 1 \end{array} \end{array}\right]\times P^{\prime }=\left[\begin{array}{cccc} x_{x{}^{\prime }} & x_{y{}^{\prime }} & x_{z{}^{\prime }} & x_{o{}^{\prime }}\\ y_{x{}^{\prime }} & y_{y{}^{\prime }} & y_{z{}^{\prime }} & y_{o{}^{\prime }}\\ z_{x{}^{\prime }} & z_{z{}^{\prime }} & z_{z{}^{\prime }} & z_{o{}^{\prime }}\\ 0 & 0 & 0 & 1 \end{array}\right]\times P^{\prime }$$

where, ***P***x, ***P***y, and ***P***z represent the direction vectors of O’X’, O’Y’, and O’Z’ in the global measurement coordinate system.

The above algorithm was used to compensate for static errors of the rotary stages. Calculate the distances *dis_z_i_* between corresponding points in the virtual point clouds and the optimized point clouds along the Z axis, which can explain the compensation effect and the reliability of the optimized rotary axis:

(11) $$dis\_z_{i}=z_{virtual\_i}-z_{optimized\_i}$$

where, *z_virtual_i_* and *z_optimized_i_* are the Z coordinates of the *i*th point in the virtual and the optimized point clouds.

**Figure 7:**
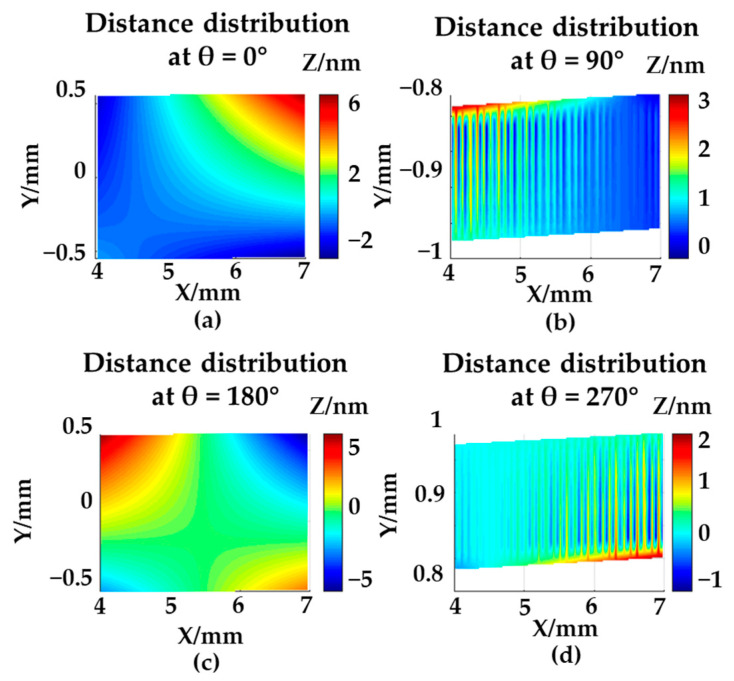
The distance distribution in the XOY plane after rotary-axis compensation in simulation: (**a**) θ = 0°; (**b**) θ = 90°; (**c**) θ = 180°; (**d**) θ = 270°.

The distance distributions after optimization are shown in Figure 7, and all distances were lower than 10 nm. The errors of the A-axis and C-axis rotary stage were compensated individually.

#### 4.2.2. Calibration and Compensation of Clamping Errors

After compensation of the static errors of two rotary stages, clamping errors were calibrated and compensated. To ensure the completeness and continuity of the whole error calibration process, the standard cylinder was also used as the calibration part for clamping errors. In simulation, the point clouds used for the calibration were also gained from the four angle 0°, 90°, 180°, and 270° on the C-axis rotary stage, but the optimization was different from the optimization above. Two points *N*_1_ (*x*_1_, *y*_1_, and *z*_1_) and *N*_2_ (*x*_2_, *y*_2_, and *z*_2_) on the rotary axis were used to determine the position and the orientation of the axis in the optimization, and the cylinder axis can be expressed as Equations (12) and (13):

(12) $$\frac{x-x_{1}}{x_{2}-x_{1}}=\frac{y-y_{1}}{y_{2}-y_{1}}=\frac{z-z_{1}}{z_{2}-z_{1}}$$

(13) $$(y_{2}+z_{2}-y_{1}-z_{1})x-(x_{2}-x_{1})y-(x_{2}-x_{1})z-x_{1}y_{2}-x_{2}y_{1}-x_{1}z_{2}-x_{2}z_{1}=0$$

The coordinates of these two points are the optimized objects. Four point clouds are in the same coordinate system and the cylinder is rotated around the C axis. During the optimization, the cylinder axis was rotated and calculated. The distance *Dis_i_* between the measured point *M_i_* (*x_i_*, *y_i_*, *z_i_*) and the cylinder axis can be calculated as Equation (14):

(14) $$Dis_{i}=\frac{\left|(y_{2}+z_{2}-y_{1}-z_{1})x_{i}-(x_{2}-x_{1})y_{i}-(x_{2}-x_{1})z_{i}-x_{1}y_{2}-x_{2}y_{1}-x_{1}z_{2}-x_{2}z_{1}\right|}{\sqrt{{\left(y_{2}+z_{2}-y_{1}-z_{1}\right)}^{2}+{\left(x_{2}-x_{1}\right)}^{2}+{\left(x_{2}-x_{1}\right)}^{2}}}$$

The optimization aims to let the point clouds coincide with the cylindrical surface. The optimized objective function is as follows:

(15) $$Obj=min(\sum_{i=1}^{n}\left|Dis_{i}-R\right|)$$

**Figure 8:**
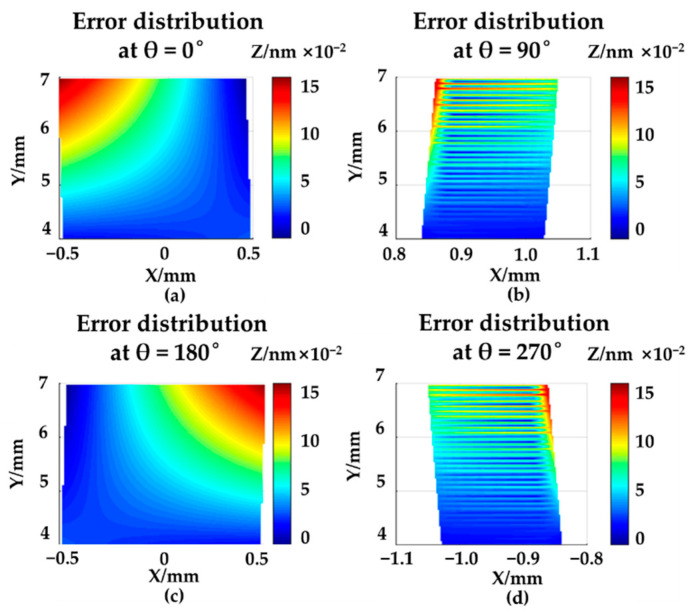
The error distribution in XOY plane after clamping-error compensation in simulation: (**a**) θ = 0°; (**b**) θ = 90°; (**c**) θ = 180°; (**d**) θ = 270°.

Based on the global optimal least square method, after optimization, the position and orientation of the cylinder axis was obtained. With the optimized cylinder axis, the error distribution was calculated based on the nominal point clouds and the measuring point clouds, as shown in Figure 8. All errors were below 1 nm, which proved that the cylinder axis was accurately positioned.

**Figure 9:**
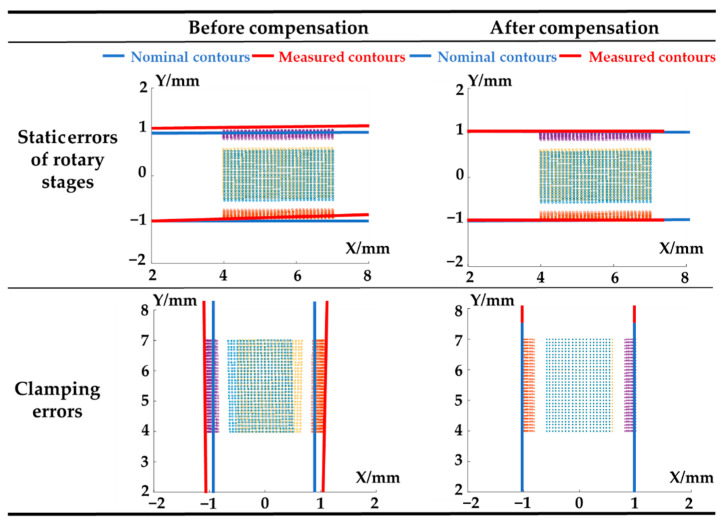
Results of the error compensation in the simulation.

The error compensation of the cradle-type five-axis measurement system mainly contains the compensation of clamping errors and errors of rotary stages based on measurement results on a standard cylinder. It can be seen from Figure 9 that influence of major error terms will be eliminated significantly via error compensation, and contours of the cylinder can be corrected after such a process. Before compensation, with the influence of static errors of rotary stages and clamping errors, the measured contours were an oblique circular cylinder and frustum of a cone, respectively. The contours changed back to nominal cylinders.

## 5. Experiments and Results

### 5.1. System Construction and Error Calibration

**Figure 10:**
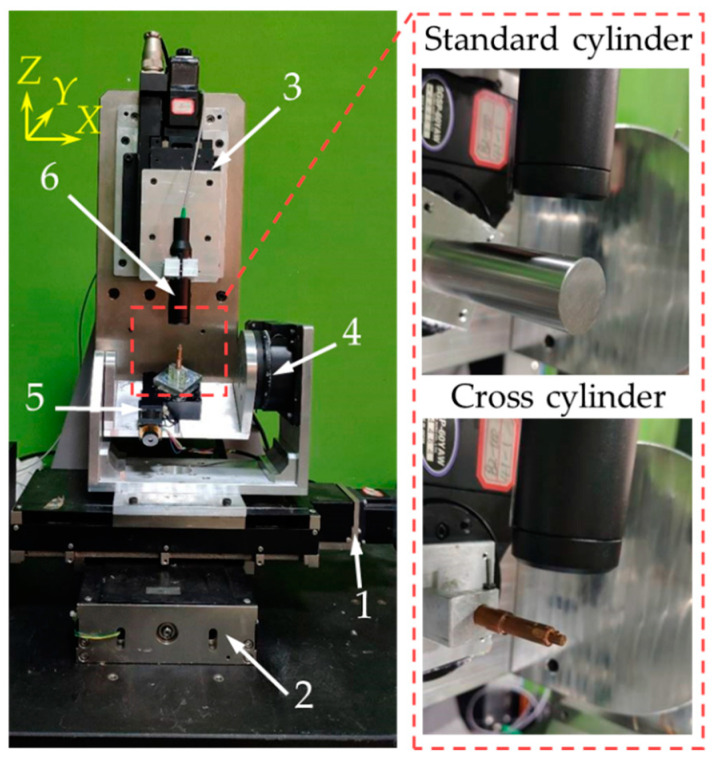
The experimental setup: 1—X axis; 2—Y axis; 3—Z axis; 4—A axis; 5—C axis; 6—probe.

The cradle-type five-axis measurement system is shown in Figure 10, and the entire experimental setup was placed on a marble air-floating base. The parameters of the hardware are shown in Table 2. The confocal probe used was produced by ThinkFocus.

Through the simulation on error calibration and compensation, the feasibility of their theories was verified. According to the simulation research, the major error terms of this self-built cradle-type five-axis measurement system with a chromatic confocal probe were calibrated, and the calibration results are shown in Table 3.

### 5.2. Measurement Results

**Figure 11:**
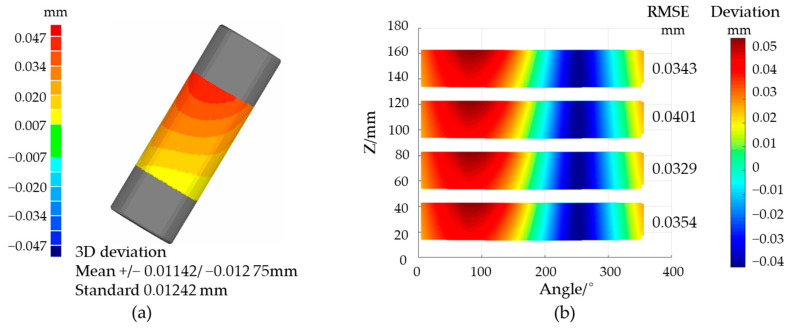
Measurement results: (**a**) the standard cylinder; (**b**) the repeatability verification.

After error compensation, the standard cylinder was measured to verify the repeatability of this system. The measurement point cloud and the ideal STL model of the measured workpieces were used for registration. After that, the vertical distance *D_i_* between the measured *i*th point and the corresponding tiny triangle surface was calculated. Suppose the number of total points is *n*, then the standard deviation σ is obtained by the calculation process of Equation (16). This standard deviation can reflect the dispersion degree of the deviation between the actual and ideal coordinates, indicating the concentration of the error distribution in a single measurement. The standard deviation in the cylinder measurement dropped from 101.27 to 12.42 μm, as shown in Figure 11a, which confirms the compensation effect. The same area of the standard cylinder was measured four times, and the performance is presented in Figure 11b. The measurement results were expanded along the angle, and the distance from each point to the cylinder axis was calculated. It can be seen from the figure that the error distribution of each measurement was basically the same, which proved that the repeatability was good.

(16) $$\sigma =\sqrt{\frac{\sum_{i=1}^{n}D_{i}^{2}}{n}}$$

**Figure 12:**
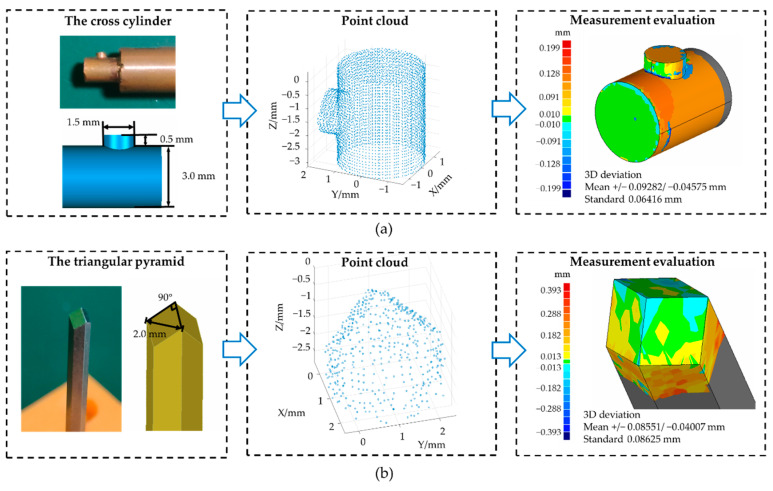
Application results on two tiny parts: (**a**) a cross cylinder; (**b**) a microtriangular pyramid.

Two kinds of typical tiny parts were selected to evaluate the overall profile measurement capability of this system, which were cross cylinders and microtriangular pyramids. These two workpieces were both manufactured by CK 6140. The orientation accuracy of this CNC machine tool was about 0.01 mm, and the reorientation accuracy was about 0.005 mm. Cross cylinders had features of rotational symmetry, and their cylindrical axes could be used to calibrate the clamping errors. As shown in Figure 12a, the cross area had a large curvature and small size, which is representative and challenging to measure. The level of detail in that area mainly depends on the sampling interval. The final point cloud was compared with the nominal 3D model. The measurement accuracy was evaluated by comparing the point cloud and nominal model. The standard deviation was 64.16 μm after compensation, which confirms the compensation effect. Besides, microtriangular pyramids have apparent contours between three sides and edges are difficult to measure. As shown in Figure 12b, a microtriangular pyramid whose three sides were all squares was measured. The cross-section of its handle was a regular hexagon. The evaluation method of measurement accuracy was the same as the process above, and the standard deviation was about 86.25 μm.

**Figure 13:**
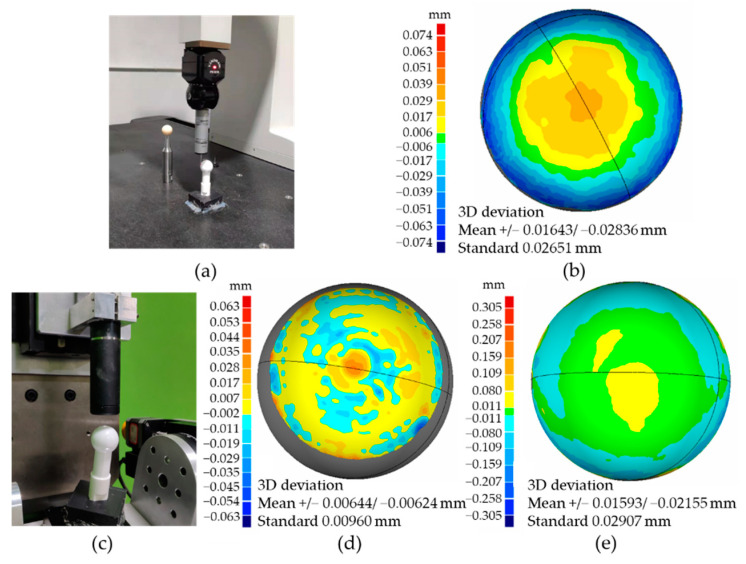
Comparation of results: (**a**) the measure process on the CMM; (**b**) measurement results (50% spherical surface) by the CMM; (**c**) the measure process on our five-axis system; (**d**) results of the same measured area (25% spherical surface) by the five-axis system; (**e**) measurement results of 70% of the spherical surface by the five-axis system.

Besides, a standard sphere was measured by a CMM (the global advantage CMM by hexagon) and our measurement system to compare measurement results, which can be seen in Figure 13a,c. Figure 13b is the evaluation of the point cloud measured by the CMM. The measured area was 50% of the entire sphere surface. The standard deviation was about 26.51 μm. Figure 13d is the measurement results of 25% of the entire surface on that sphere by our five-axis system, and the standard deviation was 9.60 μm. The error distributions were in the shape of ring bands. A total of 70% of the entire surface was also measured by this system, as shown in Figure 13e, and the standard deviation rose to 29.07 μm. Considering the accuracy of electric motion stages used, this measurement accuracy was considered reasonable. The normal radius of this standard sphere was 12.703 mm, and the results of spherical fitting with data by the CMM and our system were 12.693 mm and 12.682 mm.

## 6. Conclusions

Focusing on the difficulty of the overall profile measurements of tiny parts with complicated features, a solution using the cradle-type five-axis measurement system was proposed in this paper. We achieved the engineering implementation based on our theoretical research. It proved that this cradle-type five-axis measurement system had good value for engineering applications. Our contributions can be summarized as follows:

(1) An optical, cradle-type, non-registration point-scanning measurement method was proposed, which does not need the point cloud registration process and adapts to multiple complicated features of different sizes. This measurement system has strong flexibility.
(2) A process to identify major error terms in measurement systems and apply calibration and compensation on them was proposed. The advantages of this process are that it does not rely on any additional high-precision equipment and promotes the system’s accuracy conveniently and efficiently. This method can also be applied to correct error terms in other measurement systems with rotary axes.
(3) A five-axis experiment setup was built and tiny parts were measured in experiments. The measurement accuracy and capability of overall profile measurements were verified by measuring standard workpieces and complicated tiny parts separately. It was proved that in terms of overall profile measurement, this cradle-type five-axis measurement system had more advantages than some commercial instruments. It is worth noting that, measurement accuracy can be further improved if hardware with higher accuracy is used in the future. With the help of a premeasurement by an external measurement device, a fully automatic measurement is the next research goal.

## Figures and Tables

**Table 1:** The error classification of the five-axis measurement system.

Error Types	Specific Error Terms	Symbols	Ratios
System errors	Static errors of linear stages	*δx*, *δy*, *δz*	2.430%
	Dynamic errors of linear stages	Δ*d*	0.367%
	Static errors of rotary stages	*δθ*_1_, *δθ*_2_, Δ*x*, Δ*y*, Δ*z*	73.013%
	Dynamic errors of rotary stages	*δβ*	0.083%
Clamping errors of workpieces	Tilt errors	*δβ_w_*_1_, *δβ_w_*_2_	17.247%
	Centrifugal errors	Δ*x_w_*, Δ*z_w_*	6.860%

**Table 2:** The parameters of the five-axis measurement system.

Hardware	Travel/Range	Accuracy	Others
X/Y/Z axes	200 mm	1 μm	\
A/C axes	360°	0.004°	Surface radius of C-axis stage: 30 mm
Probe	400 μm	0.1 μm	NA: ±28°
Standard cylinder	*r*: 10 mm	Cylindricity: 14 μm	\

**Table 3:** The calibration results of the major error terms.

Error Source	Error Terms	Value (° or μm)
A axis	*δθ*_1_, *δθ*_2_	0.0741, 0.3413
	Δ*x*, Δ*y*, Δ*z*	(0.8424, −0.0012, 0.0177)
C axis	*δθ*_1_, *δθ*_2_	0.5035, −0.8466
	Δ*x*, Δ*y*, Δ*z*	(0.2906, 0.5074, 0.6875)
Workholding device	*δβ_w_*_1_, *δβ_w_*_2_	−5.6936, 2.2561
	Δ*x_w_*, Δ*z_w_*	(0.0651, −0.2498)

## Data Availability

The data are not publicly available because the data also forms part of an ongoing study.
